# Fertility preservation in female patients with hematological disorders

**DOI:** 10.1186/s12884-022-04385-9

**Published:** 2022-01-22

**Authors:** Dan Wu, Huan Shen

**Affiliations:** Reproductive Medicine Center, Peking University People’s Hospital, No. 11 of Xizhimen South Street, Xicheng District, Beijing, 100044 China

**Keywords:** Hematologic disorders, Fertility consultation, Fertility preservation, Emergency ovulation, Oocyte freezing, Frozen embryos

## Abstract

**Background:**

The aim of this study was to explore the effectiveness and safety of fertility counseling and fertility preservation using oocyte or embryo freezing prior to chemotherapy or bone marrow transplantation (BMT) in female patients with hematologic disorders.

**Methods:**

Between 2016 and 2019, 29 patients with hematologic disorders, age range 12–38 years, were given preoperative fertility counseling prior to proposed BMT. Sixteen of these patients, age range 22–38 years, chose to undergo oocyte retrieval followed by ovum or embryo freezing at our Center for Reproductive Medicine.

**Results:**

As the patients were in urgent need of chemotherapy or BMT, following the random-start controlled ovarian hyperstimulation (COH), an average of 8.2 oocytes were collected. Ten patients had an average of 6.9 oocytes frozen, while 6 patients had an average of 3.2 embryos frozen. There were no intra-operative or postoperative complications, although two patients experienced a blood transfusion reaction of the 11 transfused patients.

**Conclusion:**

For patients with hematologic disorders, oocyte or embryo freezing prior to chemotherapy or BMT may offer hope for fertility preservation in female patients. However, in order to deliver this, a standardized, feasible, and effective treatment process is needed and should include every aspect of patient selection as well as protocols for ovulation promotion, perioperative management, and postoperative observation.

## Background

According to domestic and international epidemiological data, the prevalence of malignant tumors worldwide is increasing year on year, and this trend is also seen in young people. The incidence of malignant tumors, especially those of hematological origin, is highest among people of reproductive age, including adolescents. Using leukemia as an example, the incidence of leukemia in China is approximately 3 in 100,000 patients per year. In terms of mortality from malignant tumors, leukemia ranks sixth and eighth in males and females, respectively, and first in children and adults under 35 years of age [1]. However, with the continuous progress in therapeutic technology, especially early diagnosis and effective chemotherapy, together with the development of bone marrow stem cell transplantation (BMT) technology, the survival rate of patients following treatment has improved, and the period of survival has been extended. Currently, five-year survival of patients with hematologic malignancies exceeds 80% [2].

However, while effective chemotherapy saves lives, it can also cause damage to the gonads, and for women this injury to the ovaries results in the destruction of the fertility and endocrine functions that produce sex hormones. The alkylating agents, such as cyclophosphamide, are clearly gonadotoxic, and even in patients without premature ovarian failure following chemotherapy, recovery of ovarian function has been shown to be delayed, and the response to superovulation reduced, in patients receiving these drugs as compared with those in the control group (age-matched women with male factor infertility undergoing the same treatment protocol) [3]. Knopman et al. [4] found that adolescent patients with malignancies were significantly more likely to resort to assisted reproduction techniques (mainly in vitro fertilization–embryo transfer) and had significantly lower first-time fertility rates as compared with the unaffected population, as identified through long-term follow-up. Thus, it is paramount that young patients who are planning to undergo chemotherapy or BMT that may result in damage to ovarian function obtain reproductive consultation in advance to allow them to take some protective measures for fertility preservation under the premise of the disease.

## Material and methods

From November 2016 to July 2019, 29 patients aged 12–38 years with hematological diseases underwent fertility consultation at the Reproductive Medicine Center of Peking university people’s hospital prior to proposed BMT. The patients and their families were given adequate information with an explanation of the possible impact of BMT on ovarian function and fertility. They were provided with information on fertility preservation options with an explanation of the possible risks of each method. Thus, the patients and their family members were able to make an informed choice after fully understanding and considering the information given.

Patients who chose to undergo fertility preservation were fully evaluated again, a complete treatment plan was developed, and an informed consent form was signed. Depending on the age of the patient, their ovarian reserve function, and menstrual cycle, the random-start ovarian stimulation protocol was selected. Ultrasound and serum estrogen monitoring were performed when patients were treated with controlled hyperovulation Human chorionic gonadotropin (hCG) was given in order to trigger oocyte maturation when at least two of the follicles were ≥ 18 mm, and oocyte retrieval was performed 34 to 36 h after the hCG injection. All patients were admitted to the hospital prior to oocyte retrieval, and following adequate preoperative preparation, oocyte retrieval was performed under intravenous anesthesia with no intraoperative complications. Blood hemoglobin and platelet levels are closely monitored before and after the oocyte retrieval, and red blood cell or platelet transfusions were given as necessary. Prophylactic antibiotics were also administered prior to the procedure. Once the oocytes were obtained, depending on the circumstances and wishes of the patient, the oocytes were either frozen or, following fertilization, the resulting embryos were frozen.

The study was conducted in accordance with the Declaration of Helsinki (as was revised in 2013). The study was approved by Ethics Committee of the Peking University People’s Hospital. A written consent was obtained from each patient.

## Results

**Fig. 1:**
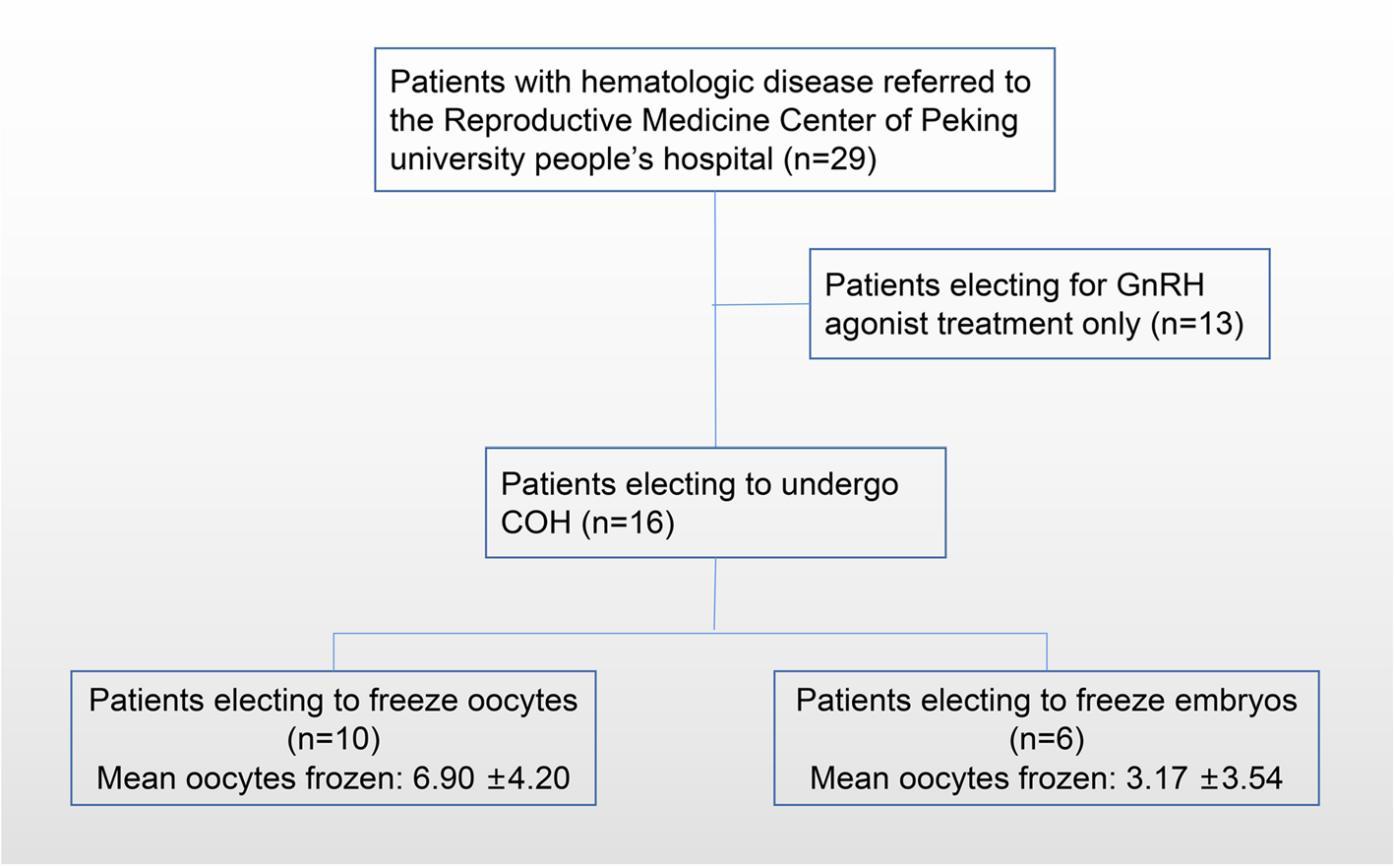
Flow chart

After thorough communication between the doctor and the patient, and careful consideration by the patients and their families, a total of 16 patients (16/29,55.2%) chose to undergo fertility preservation after the necessary physical examinations (see Table 1). After the random-start COH, an average of 8.18 ± 4.29 oocytes were collected. Ten patients underwent oocyte freezing with a mean number of 6.90 ± 4.20 frozen oocytes, and another 6 patients had a mean number of 3.17 ± 3.54 embryos frozen (see Fig. 1).

**Table 1:** General condition and ovulation promotion in patients with oocytes retrieval

Patient No	Age (years)	Diagnosis	Years of diagnosis (Year)	Times of chemotherapy (Times)	Day 1 of ovarian stimulation in the menstrual cycle (Day)	AFC (Number)	COS days (Day)	Obtained oocytes (Number)	Number of oocytes of MII (Number)	Number of frozen oocytes (Number)	Number of frozen embryos (Number)
1	25	Myelodysplastic syndrome (MDS)	4	0	17	8	5	7	1	4	
2	22	Chronic granulocytic leukemia	8	0	18	10	11	7	5	6	
3	38	Chronic aplastic anemia	15	0	2	4	5	2	1		1
4	23	Myelodysplastic syndrome (MDS)	4	0	20	10	17	9	5	5	
5	28	Acute non-lymphoblastic leukemia (M4)	2	10	3	10	9	9	6		4
6	31	Acute non-lymphoblastic leukemia (M5)	2	4	2	11	12	9	5	5	
7	23	Chronic aplastic anemia	3	0	2	10	7	18	13	13	
8	29	Acute lymphocytic leukemia	1	3	4	6	9	5	4		2
9	28	Myelodysplastic syndrome (MDS)	4 months	0	1	12	12	12	10		10
10	19	Chronic aplastic anemia	7	0	15	9	12	15	15	15	
11	27	Chronic aplastic anemia	7	0	16	10	9	7	7	7	
12	29	Acute lymphocytic leukemia	5 months	4	4	4	7	2	2		1
13	32	Acute lymphocytic leukemia	2	10	23	3	11	10	9		1
14	23	Chronic aplastic anemia	7	0	3	7	11	6	5	5	
15	25	Acute lymphocytic leukemia	5 months	4	3	5	12	9	8	8	
16	26	Acute myelogenous leukemia	3 months	2	2	5	9	4	0	1

Due to the low count of certain blood cells seen in patients with hematological disorders, blood products including red cells and platelets were transfused before and after oocyte retrieval to prevent intraoperative hemorrhage and postoperative infection. Among the 16 patients, 11 got transfusion and two experienced a transfusion reaction, of which one was a case of anaphylactic shock secondary to an allergic reaction during postoperative platelet transfusion. The patient improved following symptomatic treatment, and there were no other serious complications (see Table 2).

**Table 2:** Perioperative management of patients undergoing oocyte retrieval

Patient No	Blood routine test before oocyte retrieval			Blood routine test after oocyte retrieval			Blood transfusion			Complication
	WBC (×10^9^/L)	Hb (g/L)	PLT (×10^9^/L)	WBC (×10^9^/L)	Hb (g/L)	PLT (×10^9^/L)	Pre-operative	Intra-operative	Post-operative	
1	2.60	77	15	1.90	63	118		Platelet 1 U	Concentrated red blood cells 200 ml	
2	10.20	116	240	9.30	108	222				
3	2.30	78	41	1.30	71	16			Leukocyte-removed suspension red blood cells 2 IU	
4	1.5	83	16	0.90	68	76	Platelet 1 U		Platelet 1 U	
5	3.30	67	54	3.90	73	62	Platelet 1 U		Platelet 1 U	
6	3.20	68	43	3.30	70	68	Platelet 1 U		Platelet 1 U	
7	2.60	99	31	4.10	81	86			Platelet 1 U	Anaphylactic shock occurred during platelet transfusion after the operation, which improved after symptomatic treatment
8	1.32	73	64	1.32	70	67	Platelet 1 U			
9	3.80	98	51	7.00	100	81	Platelet 1 U			Skin rash occurred during platelet transfusion before the operation, which improved after symptomatic treatment
10	2.30	75	66	2.20	68	66	Platelet 1 U		Concentrated red blood cells 200 ml	
11	2.34	68	27	1.66	64	57	Platelet 1 U + Concentrated red blood cells 200 ml		Concentrated red blood cells 200 ml	
12	5.24	114	167	5.80	124	239				
13	3.61	122	285	3.84	132	294				
14	4.7	90	24	4.6	82	96	Platelet 2 U			Skin pruritus during platelet transfusion, get better upon stop transfusion
15	7.00	111	180	8.10	113	151				
16	5.11	131	180	5.34	128	173				

Of the 13 patients who underwent fertility counseling but did not proceed to oocyte retrieval, 2 patients were only 12 and 16 years old. Due to their young age and immature gonadal development, they were not suitable for ovulation promotion and oocyte retrieval surgery. Instead, the possibility of ovarian tissue freezing in order to preserve fertility was explained to the patient, but after considering this option the families refused [5]. The remaining 11 patients did not have the opportunity for ovulation promotion due to the urgency of their proposed BMT. However, following fertility counseling, all patients chose to protect ovarian function with gonadotropin-releasing hormone (GnRH) agonist injections during chemotherapy [6].

## Discussion

### Fertility consultation

The Institute of Hematology at the Peking University People’s Hospital is one of the largest comprehensive hematology institutes in China, with more than 370 beds, including 260 beds for stem cell transplantation. It is the largest hematopoietic stem cell transplantation (HSCT) center in Asia and one of the top five in the world, completing nearly 800 hematopoietic stem cell transplants each year, representing approximately one quarter of the total number of similar transplants in China. However, even with such a large volume of hematology treatments, few patients can visit the fertility center for fertility counseling prior to chemotherapy or transplantation.

Due to the specificity of hematologic diseases, in addition to acute and malignant diseases, some non-malignant diseases, such as myelodysplastic syndrome and aplastic anemia, also require HSCT. Systemic chemotherapy prior to HSCT in the treatment of these diseases has been shown to seriously impair fertility [7]. However, these patients do not require immediate chemotherapy as in the case of those with acute, malignant disease, and for most there will be a long period of time from making the decision to have a BMT to starting treatment. In contrast, all of the patients in this study presented to the Fertility Center during routine physical examinations prior to BMT and at a time that was very close to the date the patient was due to start treatment. Consequently, some patients may lose the opportunity to benefit from fertility preservation due to timing issues. Therefore, if fertility counseling can be integrated into routine clinical practice, the majority of patients with non-acute, non-malignant diseases will have time to comfortably consider fertility issues and may have sufficient time to undergo fertility preservation. In our study, 16 patients selected fertility preservation in all 29 patients, and 11 patients didn’t select due to the urgency of their proposed BMT.

Fertility counseling should include a comprehensive fertility assessment, and ovarian reserve function in each patient should be evaluated, regardless of whether or not the patient has chemotherapy. As the timing of visits in the study patients was random, and it was difficult to assess the antral follicle count, anti-Mullerian hormone was considered to be more appropriate. Following evaluation, the most suitable plan for preserving fertility could be provided based on the age, marital status, ovarian function, primary disease, and the wishes of the patient and family.

Currently, for adults and postpubertal patients, oocyte or embryo cryopreservation is an established method. If legally permitted and necessary, they may also consider using donor sperm or gestational carriers If they don’t have enough treatment time, ovarian tissue cryopreservation is offered as an experimental technique. For prepubertal girls, ovarian tissue cryopreservation is the only option. As for ovarian protection, there is insufficient evidence regarding the effectiveness of GnRH agonist in fertility preservation [8].

### Selection of ovulation promotion protocol

For the majority of the patients in this study, the random timing of their visits and the urgency of the proposed chemotherapy or BMT meant that they could not be treated according to the conventional clinical protocol of controlled ovarian hyperstimulation (COS) and were therefore scheduled for ovulation as soon as possible according to their menstrual cycle, i.e., using the random-start COS.

Generally, when the patient was in the late follicular stage (i.e., after the seventh day of menstruation, with the dominant follicle > 13 mm and/or serum progesterone (P) < 2 ng/ml), ovulation-promoting drugs were given as appropriate to promote dominant follicle growth as soon as possible. Oocyte maturation was triggered with hCG or a GnRH agonist when the dominant follicle had developed to 18 mm, and this was followed by luteal phase COS 2–3 days later.

If the patient was in the luteal phase at the time of their visit, i.e., *P* > 3 ng/ml, ovarian stimulation could be started immediately and without the application of GnRH antagonists. If the patient was in the mid-luteal phase, GnRH antagonists could be administered to induce luteal atrophy, after which the serum P level would drop and menstruation would occur 2–4 days later. Thus, the COS might be started earlier than by waiting for natural menstruation [9].

### Safety of oocytes retrieval

Due to the clinical characteristics of patients with hematological disease, the risk of intraoperative and postoperative hemorrhage and infection is greatly increased. Therefore, a detailed preoperative evaluation of routine blood tests should be performed. Generally, it is safer to perform surgery with a hemoglobin level of at least 80 g/L and a platelet count of at least 50 × 10^9^/L [10]. A relatively low blood count should be corrected by transfusion of red blood cells and platelets before, during, and after surgery, while remaining vigilant of the possibility of transfusion reactions. These patients have a high incidence of anaphylaxis due to multiple previous transfusions. These patients also have a higher risk of infection than the normal population, so intravenous antibiotics should be given prophylactically before and after oocyte retrieval. In summary, oocyte retrieval in patients with hematological disease may not be absolutely contraindicated, and the safety of oocyte retrieval can be ensured by component blood transfusion in cases of complete blood cell depletion.

### Culture of the immature oocytes

The maturity of the oocytes obtained from these patients may also vary because of the random timing of ovulation initiation and the possible lack of follicle uniformity. In order to maximize the chances of future fertility for the patient, the immature oocytes obtained should be cultured in vitro and frozen or inseminated when they became mature. In this study, seven oocytes were retrieved from the first patient, only one of which was a stage MII mature oocyte. Thus, the remaining immature oocytes were cultured in vitro and three more oocytes were matured 4 hours later. A total of four oocytes were eventually frozen for this patient. In a study that included 248 breast cancer patients before neoadjuvant chemotherapy, a mean number of 6.4 ± 0.3 mature oocytes were cryopreserved after IVM [11]. The first livebirth following vitrification of in vitro matured oocytes has been reported in 2020 [12].

### Ovum freezing

Many young women with hematological disorders are either unmarried or not in a stable relationship. Therefore, if embryo freezing was the only option for fertility preservation, this opportunity would not be available to them. Consequently, the possibility of oocyte freezing will undoubtedly give them hope. Vitrification is a very effective method for freezing oocytes, is significantly better than that of slow programmed freezing, and is associated with a greater survival rate of frozen oocytes [13]. With the gradual maturation of oocyte freezing technology, the recovery rate, fertilization rate, and clinical pregnancy rate using frozen oocytes have all improved significantly [14, 15]. It has been reported that following fertilization of the thawed mature MII oocytes to form embryos, the pregnancy rate after single embryo transfer may reach 32–65%, the live birth rate could be more than 50%, and the rate of congenital anomalies in the neonate is similar to that of natural pregnancy [16]. A recent report of the largest series to date, 1468 women undergoing elective oocyte cryopreservation for non-oncologic reasons, of whom 137 returned to use them, showed that pregnancy rates were age-dependent and the optimal number of stored MII oocytes was at least 8–10 [17].

### Summary and outlook

The 2012 revision of the US Clinical Practice Guidelines for Oncology states that the treatment of modern oncology is not the sole responsibility of oncology, but rather the efforts of clinical oncology, radiation oncology, gynecological oncology, urology, hematology, pediatric oncology, surgery, and other disciplines should combine to provide more protection for the treatment and prognosis of patients with tumors [18]. With the establishment of oncologic assisted reproduction techniques, medical practitioners have recognized the importance of fertility preservation in the long-term prognosis and quality of life of patients. A new edition of the American Society of Clinical Oncology guidelines for survivorship in patients with tumors, published in the Journal of Clinical Oncology in 2013, recommends that as well as discussing the success rate of anti-cancer treatment, if there is a risk of infertility, then fertility preservation measures should also be discussed with those patients with fertility needs and their families before initiating treatment [19].

It is suggested that as the technology associated with the treatment of disease has improved, fertility preservation now receives more and more attention. However, due to the long duration of treatment of the primary disease, and the post-operative recovery period in the patient, there is a paucity of large-scale reports of pregnancy outcomes following fertility preservation in patients with hematological diseases, and further studies and follow-up are needed.

## Data Availability

The datasets used and/or analysed during the current study available from the corresponding author on reasonable request.

## Abbreviations

BMT: Bone marrow transplantation
hCG: Human chorionic gonadotropin
GnRH: Gonadotropin-releasing hormone
HSCT: Hematopoietic stem cell transplantation
COS: Controlled ovarian hyperstimulation
